# Supplementary material for: Detecting and phasing minor single-nucleotide variants from long-read sequencing data
Source: Nat Commun. 2021 May 24;12:3032. doi: 10.1038/s41467-021-23289-4 (PMC8144375; doi:10.1038/s41467-021-23289-4)
Supplement: Supplementary file 1 — Supplementary Information [file 41467_2021_23289_MOESM1_ESM.pdf]

Supplementary Information Feng *et al.*

Detecting and phasing minor single-nucleotide variants from long-read sequencing data

Supplementary Figures

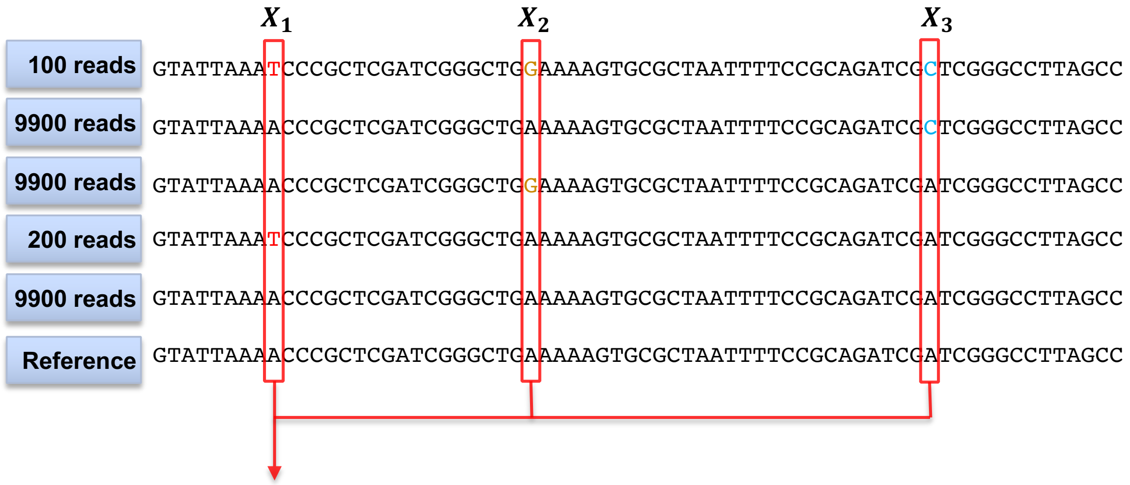

$$Pr(X_1 = T) = 300 / 30000 = 0.01$$
$$Pr(X_1 = T \mid X_2 = G) = 100 / 10000 = 0.01$$
$$Pr(X_1 = T \mid X_3 = C) = 100 / 10000 = 0.01$$
$$Pr(X_1 = T \mid X_2 = G, X_3 = C) = 100 / 100 = 1$$

Supplementary Figure 1: **Fixing the number of dependent loci might lead to false negatives.** It is difficult to detect X_1 by its marginal substitution rate or its conditional substitution rate given X_2 or X_3. However, X_1 has a large conditional substitution rate given the combination of X_2 and X_3.

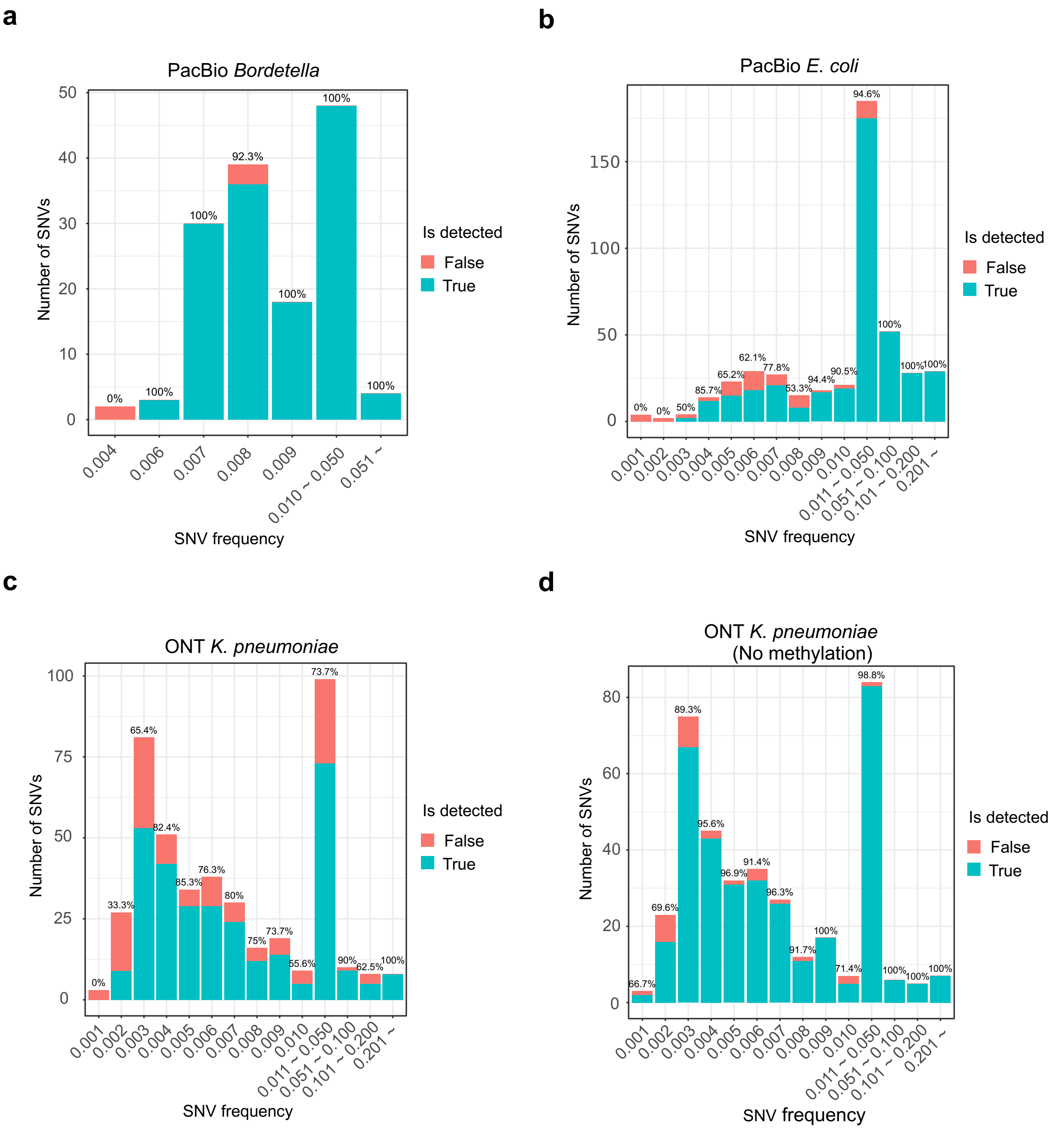

Supplementary Figure 2: **Sensitivity of SNV detection under different SNV frequencies.** The Y-axis is the number of detected and undetected SNVs under FDR (False Discovery Rate) less than 1%, and X-axis is SNV frequency. **a** The detection sensitivity stratified by SNV frequency on the PacBio *Bordetella* data. **b** The detection sensitivity stratified by SNV frequency on the PacBio *E. coli* data. **c** The detection sensitivity stratified by SNV frequency on the ONT *K. pneumoniae* data. **d** The detection sensitivity stratified by SNV frequency on the ONT *K. pneumoniae* data with methylated loci masked. Source data are provided as a Source Data file.

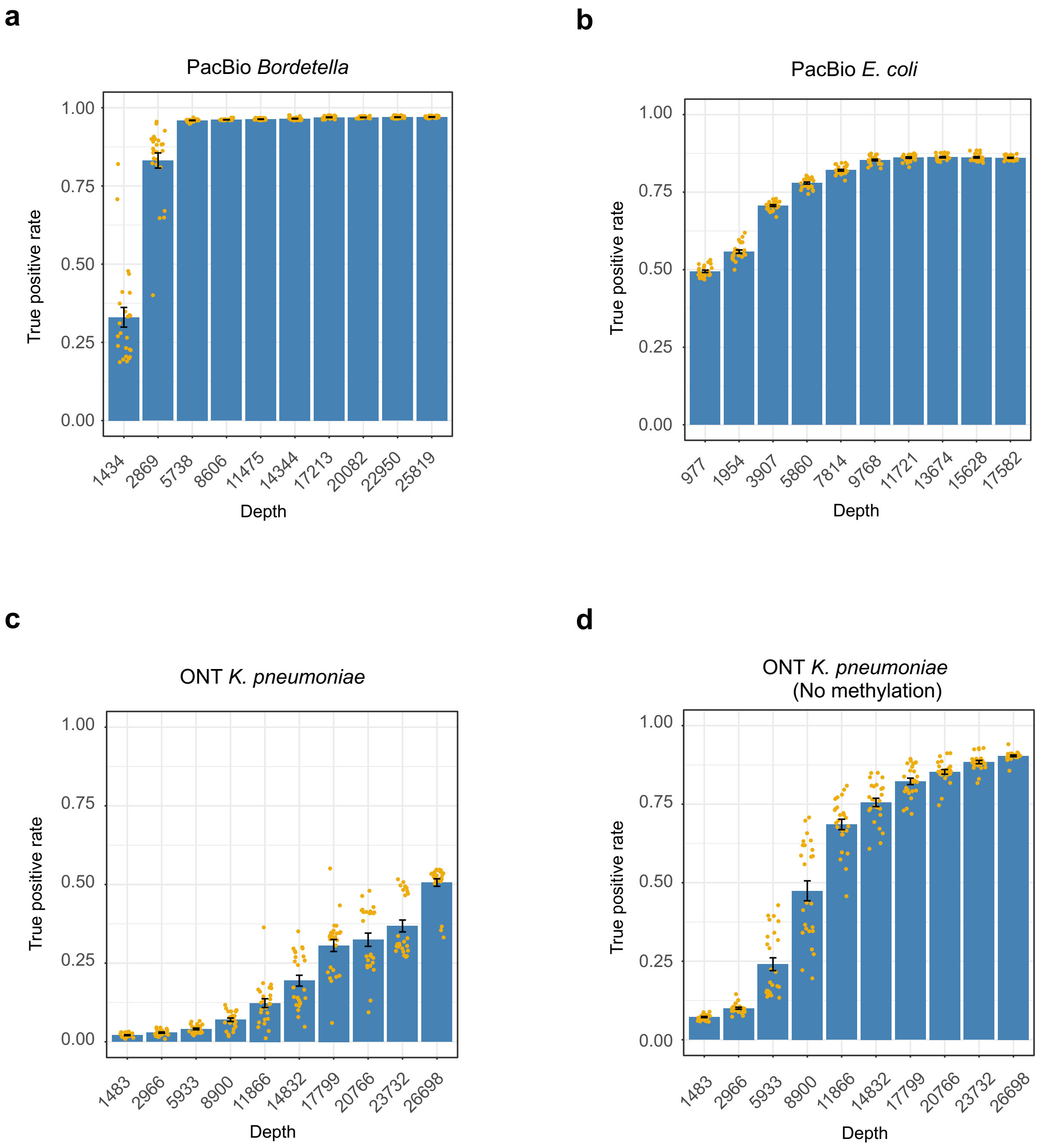

Supplementary Figure 3: **Sensitivity of SNV detection under different sequencing depths.** The Y-axis is true positive rate under FDR (False positive rate) less than 1%, and X-axis is sequencing depth obtained by subsampling the reads. The height of each bar is the average true positive rate by subsampling the reads 25 times. Each error bar is (mean - standard variance, mean + standard variance). **a** The true positive rates on the PacBio *Bordetella* data. **b** The true positive rates on the PacBio *E. coli* data. **c** The true positive rates on the ONT *K. pneumoniae* data. **d** The true positive rates on the ONT *K. pneumoniae* data with methylation masked. Source data are provided as a Source Data file.

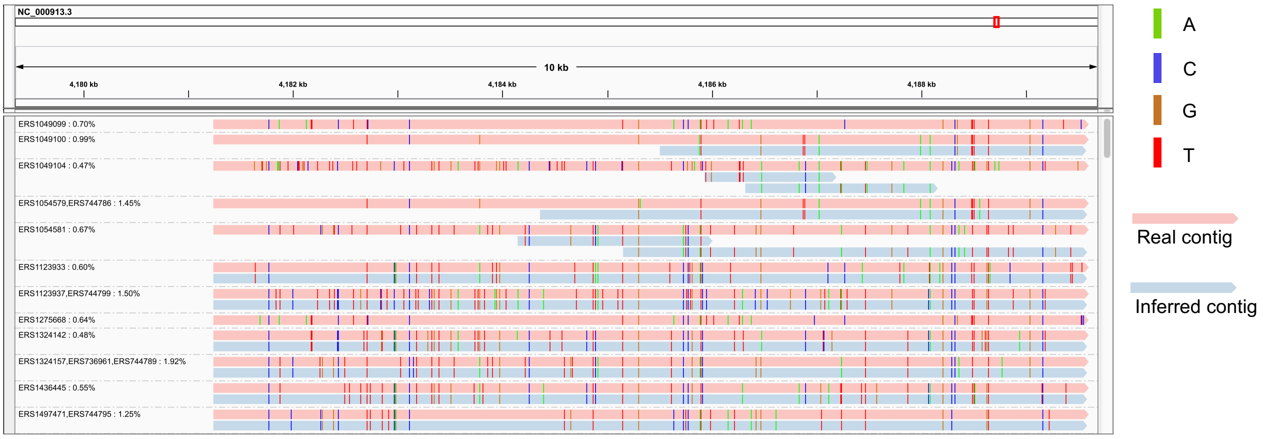

Supplementary Figure 4: **The IGV snapshot of the contigs inferred by iGDA from the PacBio *E. coli* data.** Each contig is grouped with its closest real contig. Some contigs are not shown in this figure due to size limit of IGV snapshot. Source data are provided as a Source Data file.

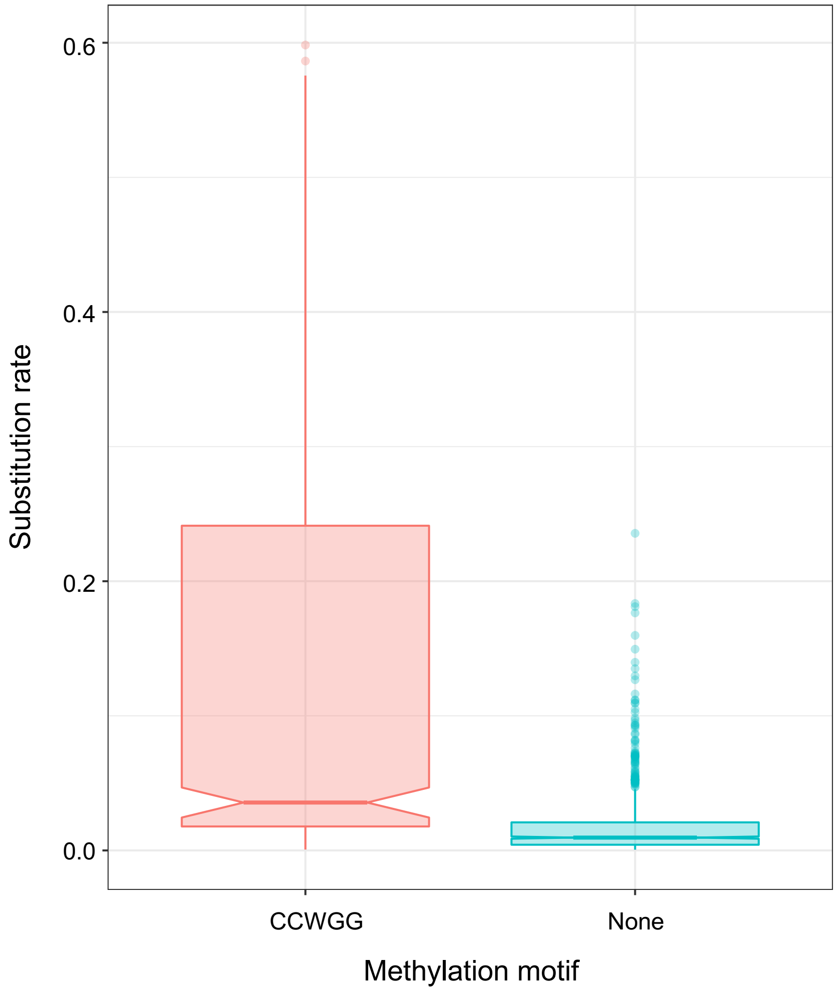

Supplementary Figure 5: **Substitution rates of the loci with methylation motif.** The figure shows the substitution rates of randomly selected 1,000 CCWGG motif loci and 1,000 non-motif loci on a individual ONT dataset, SRR8054586. In each boxplot, the center line is the median, the notches are the median +/- 1.58× IQR / $\sqrt{n}$, where IQR is the range between the 75% quantile and 25% quantile and n is the sample size. The upper and lower end of each box are the 75% quantile and 25% quantile respectively, and the ends of the vertical line in the center of each box are min(75% quantile $+ 1.5 \times$ IQR, maximum) and max(25% quantile $- 1.5 \times$ IQR, minimum) respectively. The data points out of the range of the vertical lines are shown in the box plots. Source data are provided as a Source Data file.

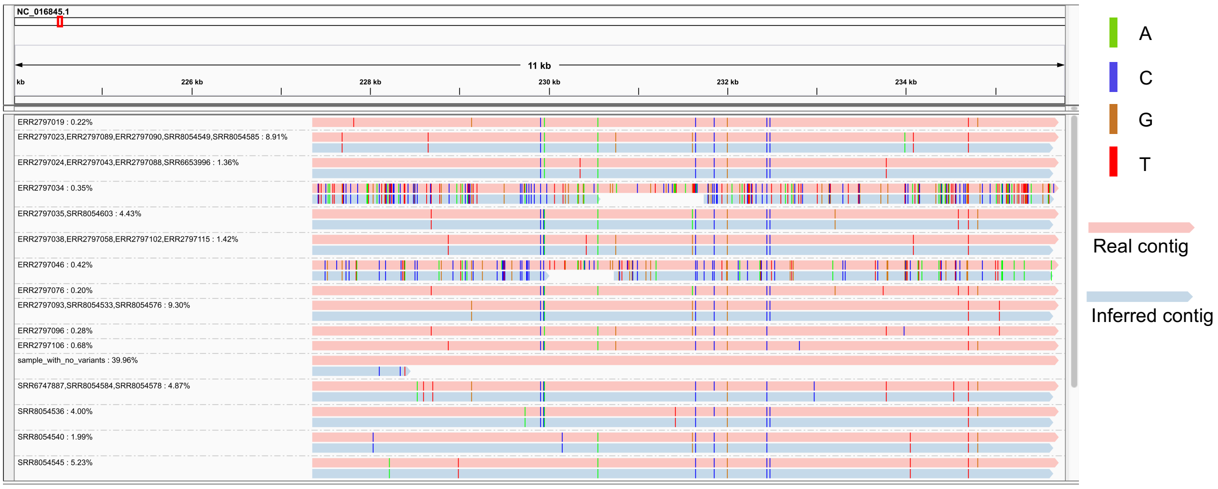

Supplementary Figure 6: **The IGV snapshot of the contigs inferred by iGDA from the ONT *K. pneumoniae* data.** Each contig is grouped with its closest real contig. Some contigs are not shown in this figure due to size limit of IGV snapshot. Source data are provided as a Source Data file.

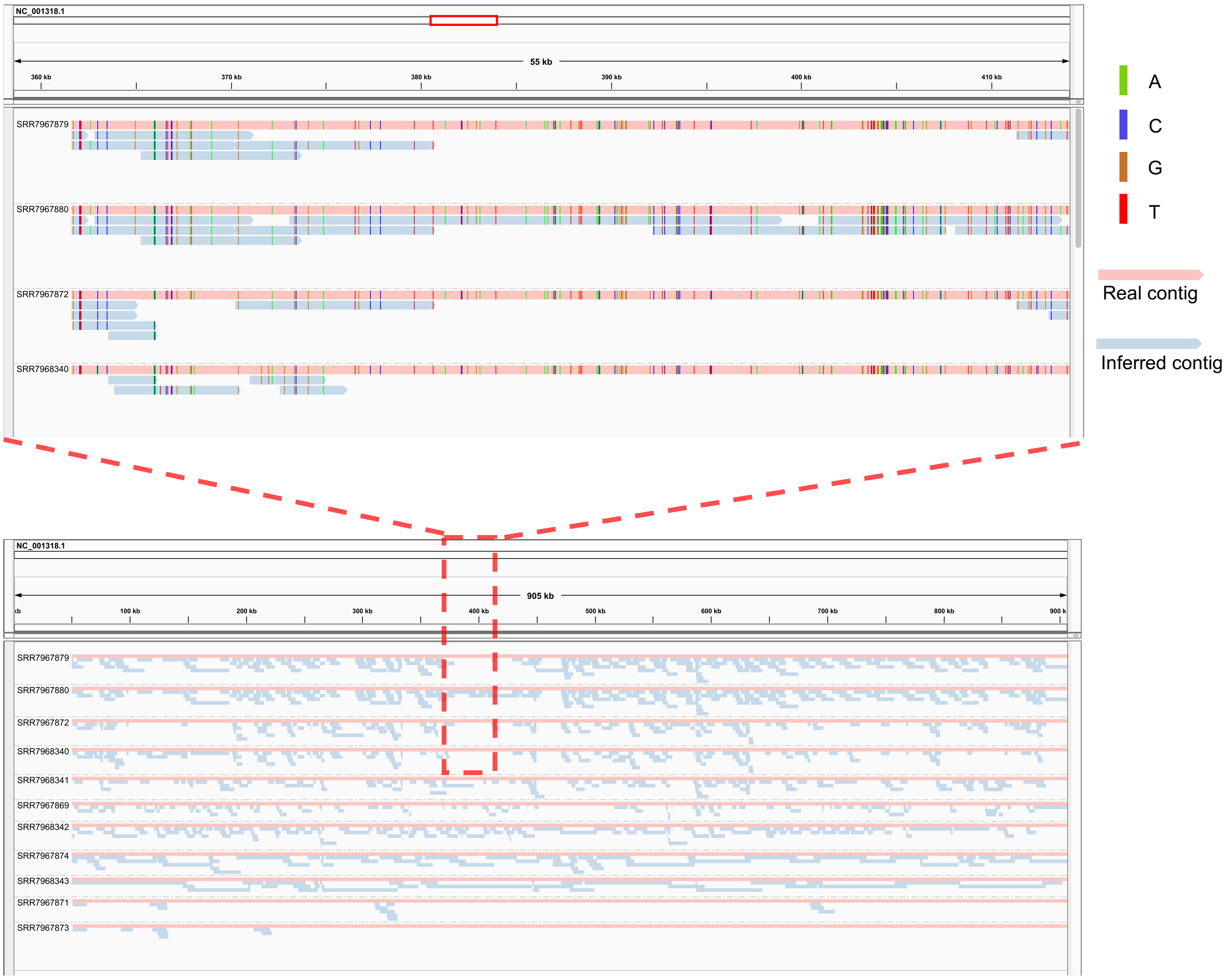

Supplementary Figure 7: **Missed regions due to highly similar strains.** The lower panel shows the contigs reported by iGDA. The upper panel shows the contigs in region [363553, 414384]. Each contig is grouped with its closest real contig. Source data are provided as a Source Data file.

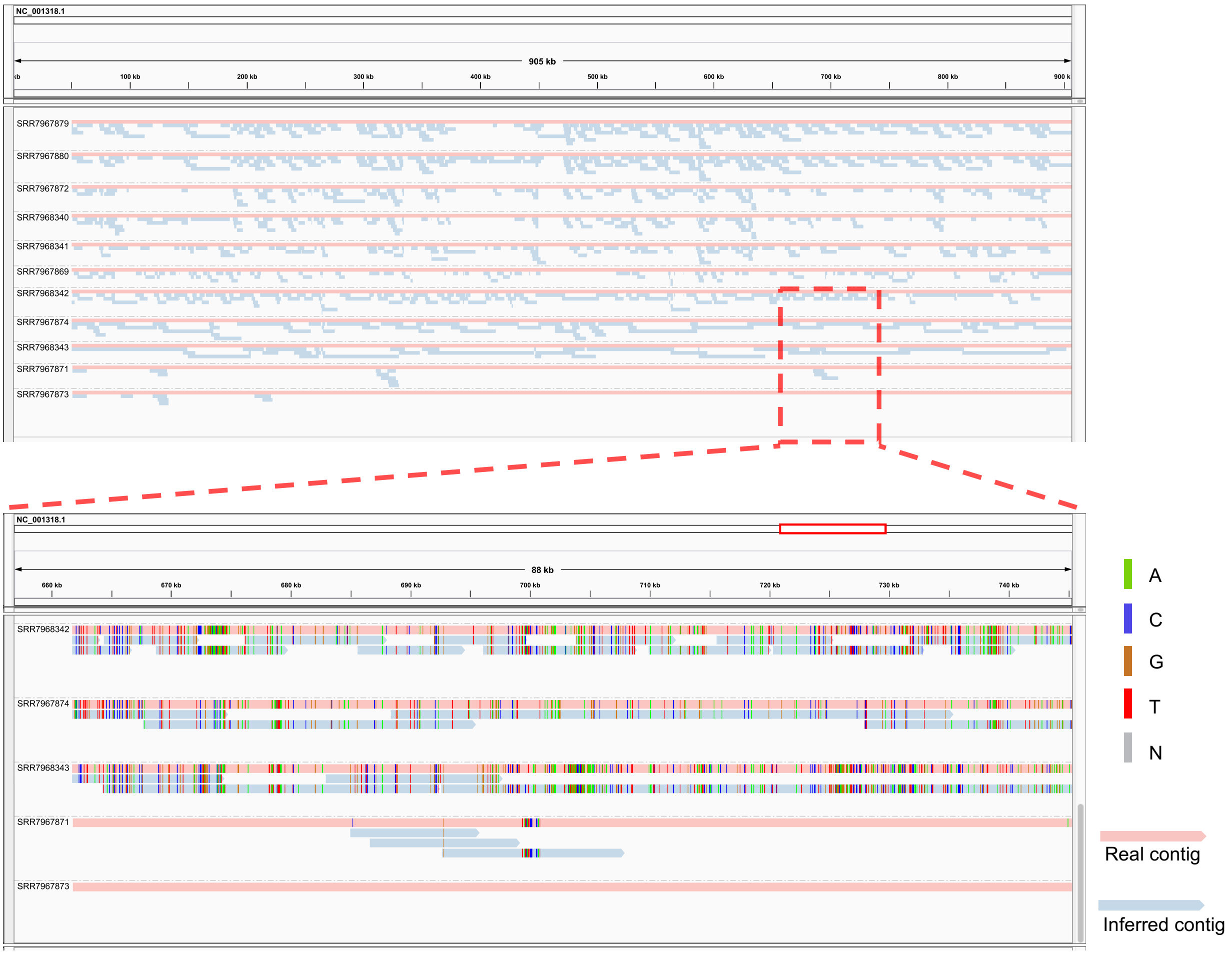

Supplementary Figure 8: **Missed regions that have no SNV compared to the reference genome.** The upper panel is the contigs reported by iGDA. The lower panel is the contigs in region [656843, 745718]. Each contig is grouped with its closest real contig. Samples SRR7967871 and SRR7967873 have several large missed regions, which have no SNV compared to the reference genome. Source data are provided as a Source Data file.

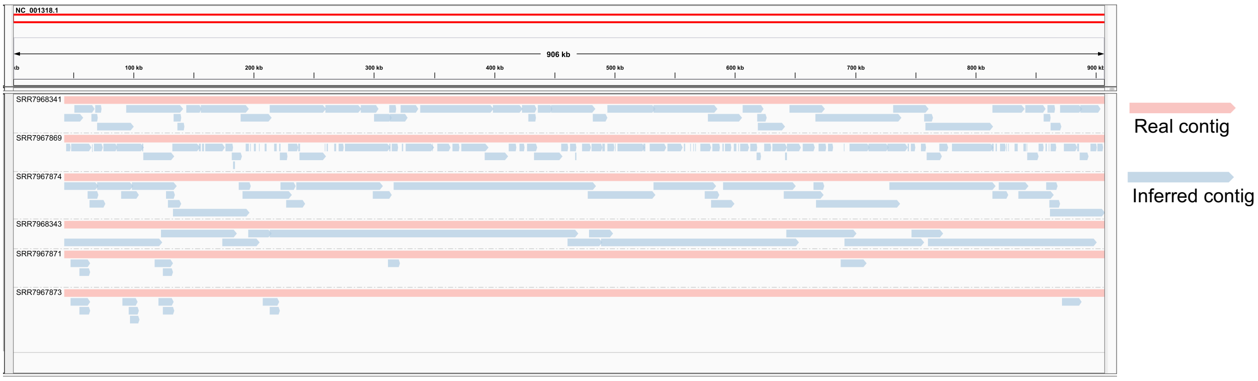

Supplementary Figure 9: **The IGV snapshot of contigs on the metagenomic data by removing highly similar strains.** Source data are provided as a Source Data file.

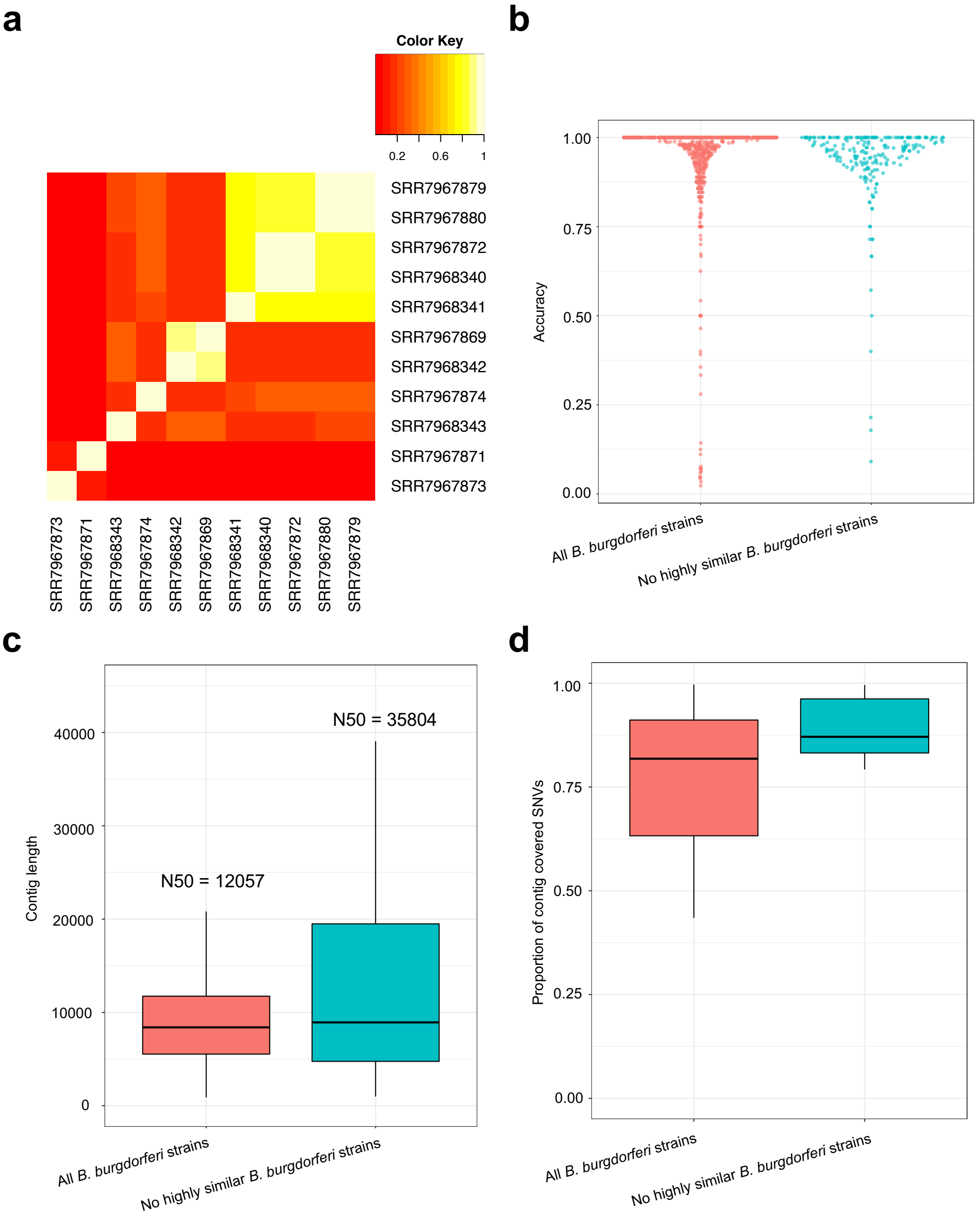

Supplementary Figure 10: **Impact of highly similar strains on the performance of iGDA. a** Heatmap of Jaccard index of the samples. **b** Sina plot of accuracy of the iGDA inferred contigs. **c** Box plots of contig length reported by iGDA. In each box plot, the center line is the median, the upper and lower end of each box are the 75% quantile and 25% quantile respectively, and the ends of the vertical line in the center of each box are min(75% quantile + 1.5 × IQR, maximum) and max(25% quantile − 1.5 × IQR, minimum) respectively. IQR is the range between the 75% quantile and 25% quantile. The statistics in the boxplots are derived from 753 contigs for "All *B. burgdorferi* strains" and 233 contigs for "No highly similar *B. burgdorferi* strains". **d** Box plots of proportion of SNVs covered by the contigs for each strain. The definition of box plot elements and number of contigs are the same as subfigure **c**. Source data are provided as a Source Data file.

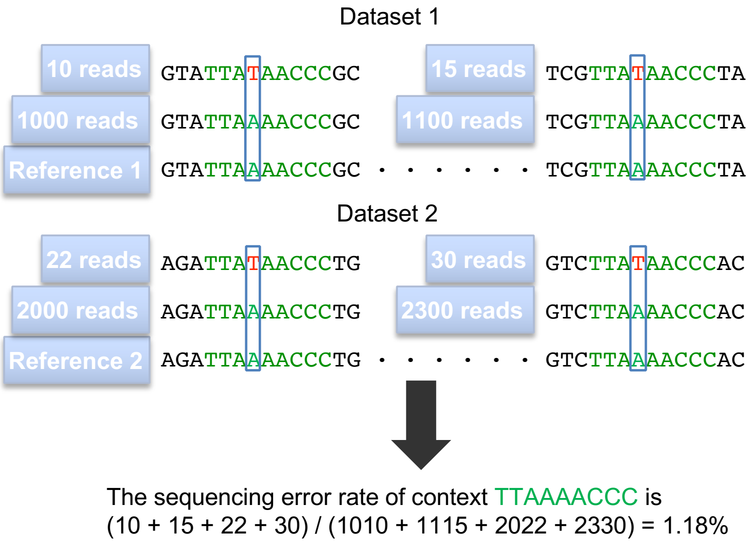

Supplementary Figure 11: **Predict sequencing error rate by sequence context.** The sequence context of a locus is the concatenated sequence of one upstream homopolymer, the homopolymer containing the very locus, and one downstream homopolymer. The features of the sequence context are the identity and number of bases in these homopolymers. The feature vector of sequence context, TTAAAACCC, is (2, T, 4, A, 3, C).

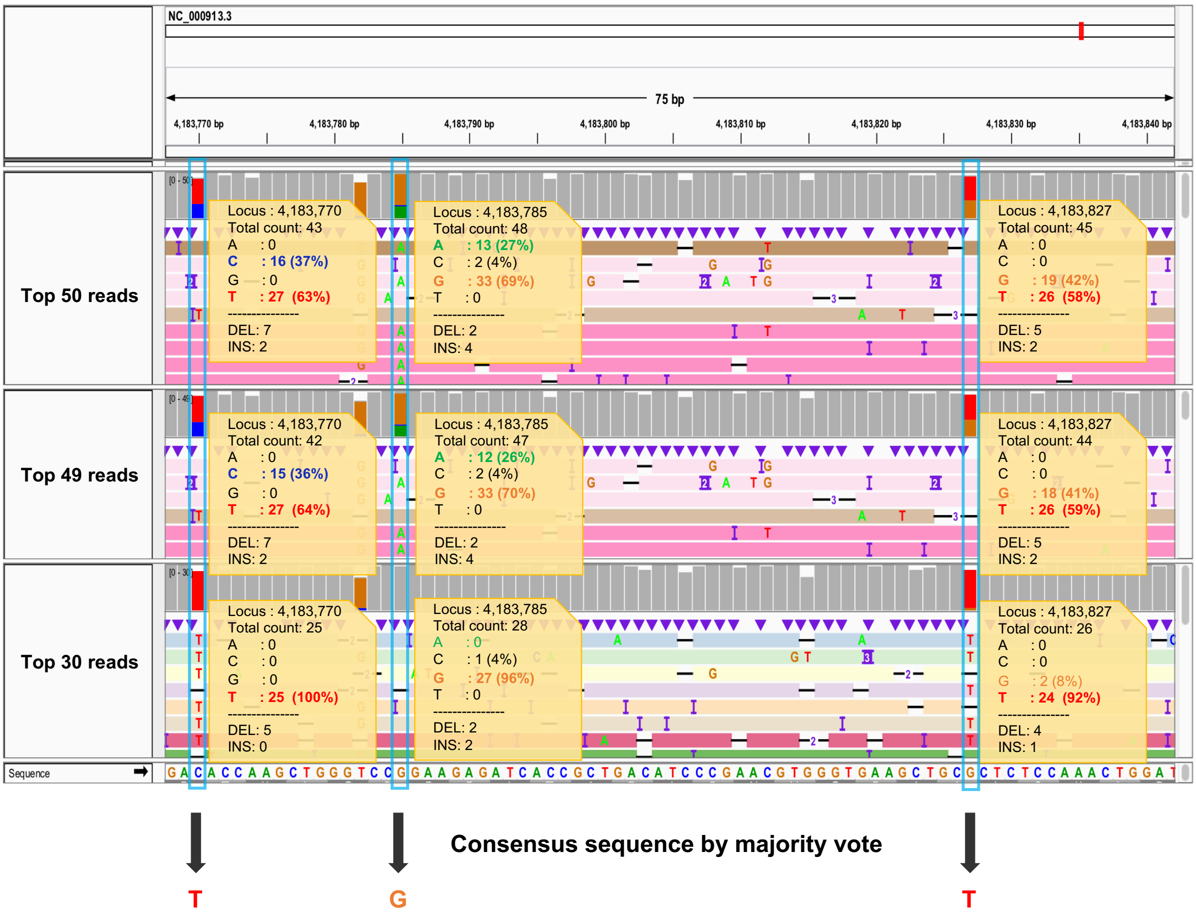

Supplementary Figure 12: **An example of ANN algorithm.** Each read in the IGV snapshot is colored according to its Jaccard Index with the seed read.

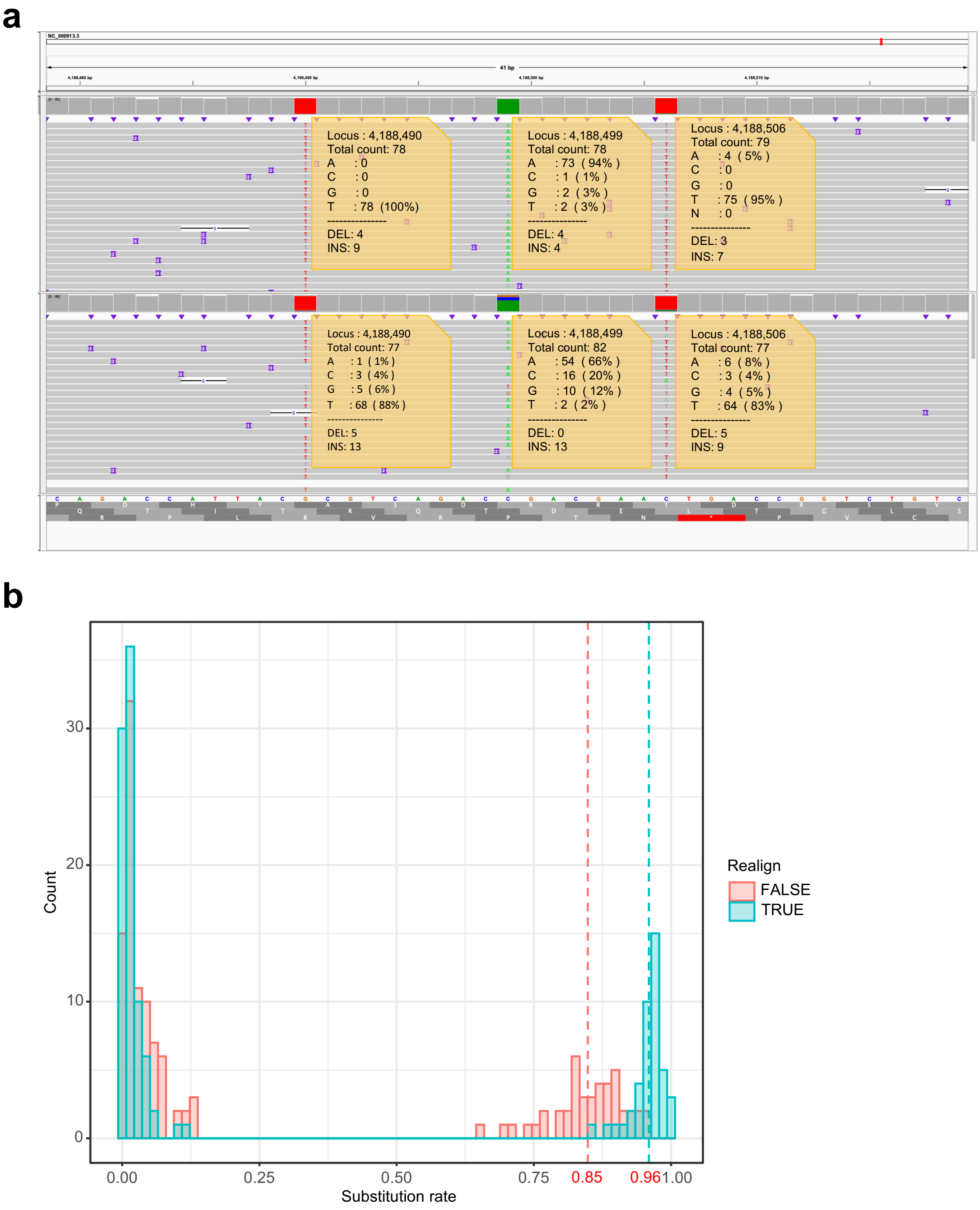

Supplementary Figure 13: **Reference bias. a** The IGV snapshot of PacBio sequencing data of an individual *E. coli* sample that is presumably homogeneous. The bottom track is the alignment to the original reference genome and the upper track is the alignment to the modified reference genome where the base at locus 4,188,499 is changed from C to A. **b** The distribution of substitution rate before and after realignment based on 261 substitutions. Source data are provided as a Source Data file.

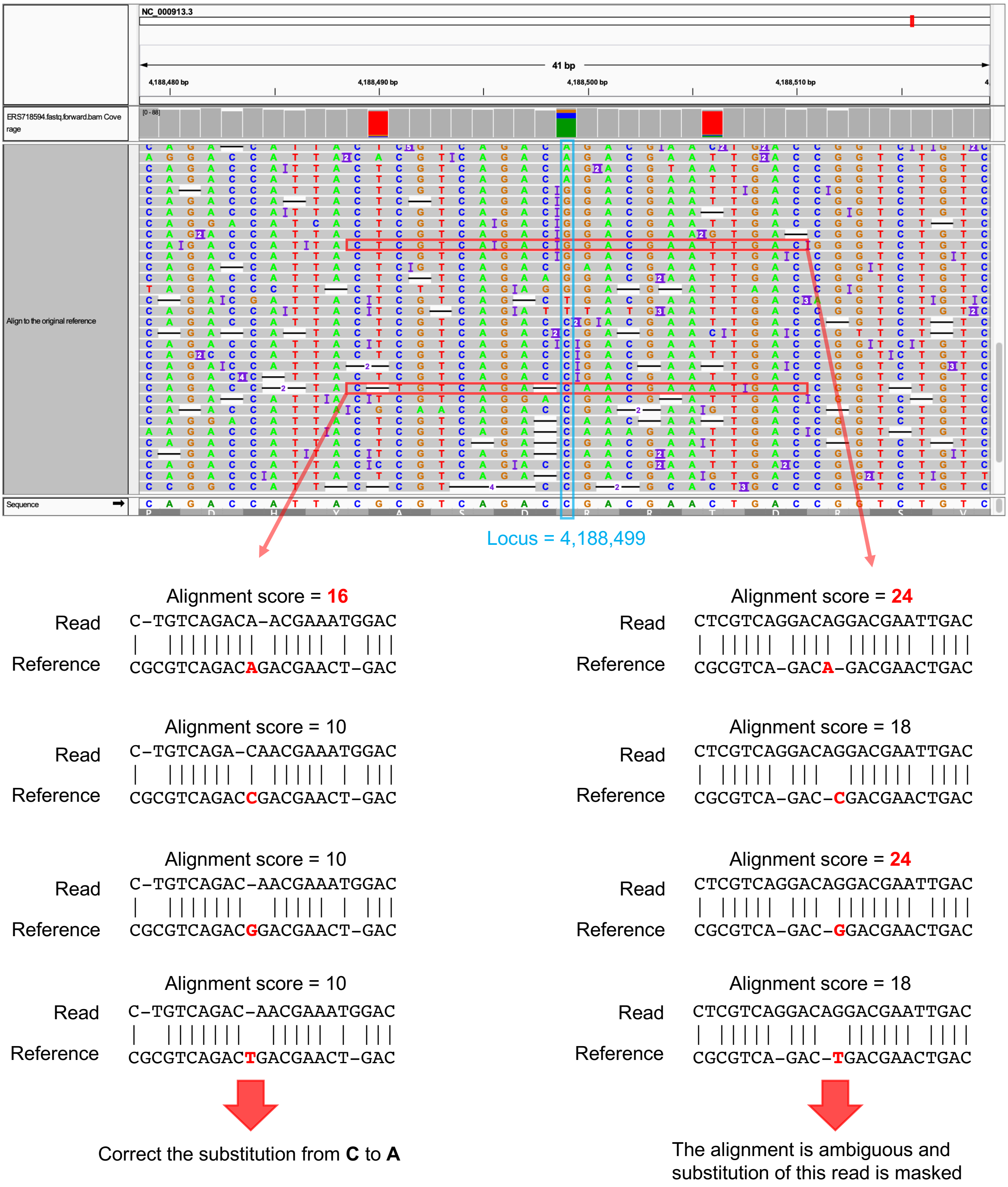

Supplementary Figure 14: **Correcting reference bias by realignment.**

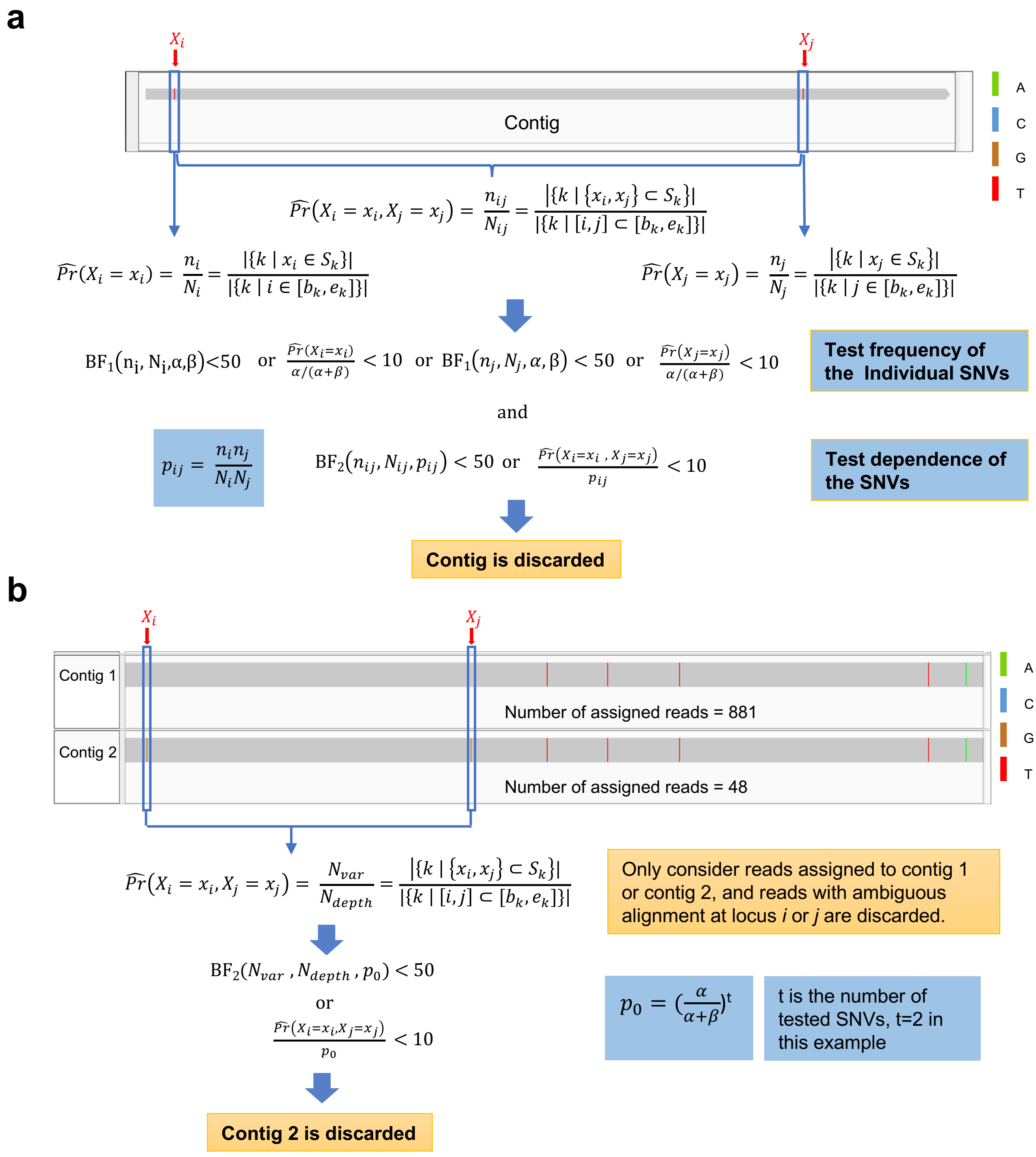

Supplementary Figure 15: **Filtering contigs.** Bayes factors $\mathrm{BF}_1(n, N, \alpha, \beta) = \frac{\mathrm{Beta}(n+1, N-n+1)\mathrm{Beta}(\alpha,\beta)}{\mathrm{Beta}(n+\alpha, N-n+\beta)}$, and $\mathrm{BF}_2(n, N, p) = \frac{\mathrm{Beta}(n+1, N-n+1)}{n^p(N-n)^{(1-p)}}\left(\ 1 - \int_0^p t^n(1-t)^{N-n}dt\ \right)$. α and β are estimated by fitting a Beta distribution on subsitution rate obtained from the independent data in Table S4. $\alpha = 1.332824, \beta = 89.04769$ for PacBio data, and $\alpha = 0.646625, \beta = 21.90139$ for ONT data. **a** Discarding a contig if the frequencies of all its SNVs are not significantly higher than error rate and its SNVs are independent. **b** Discarding a contig if it is not significantly different from its similar contig.

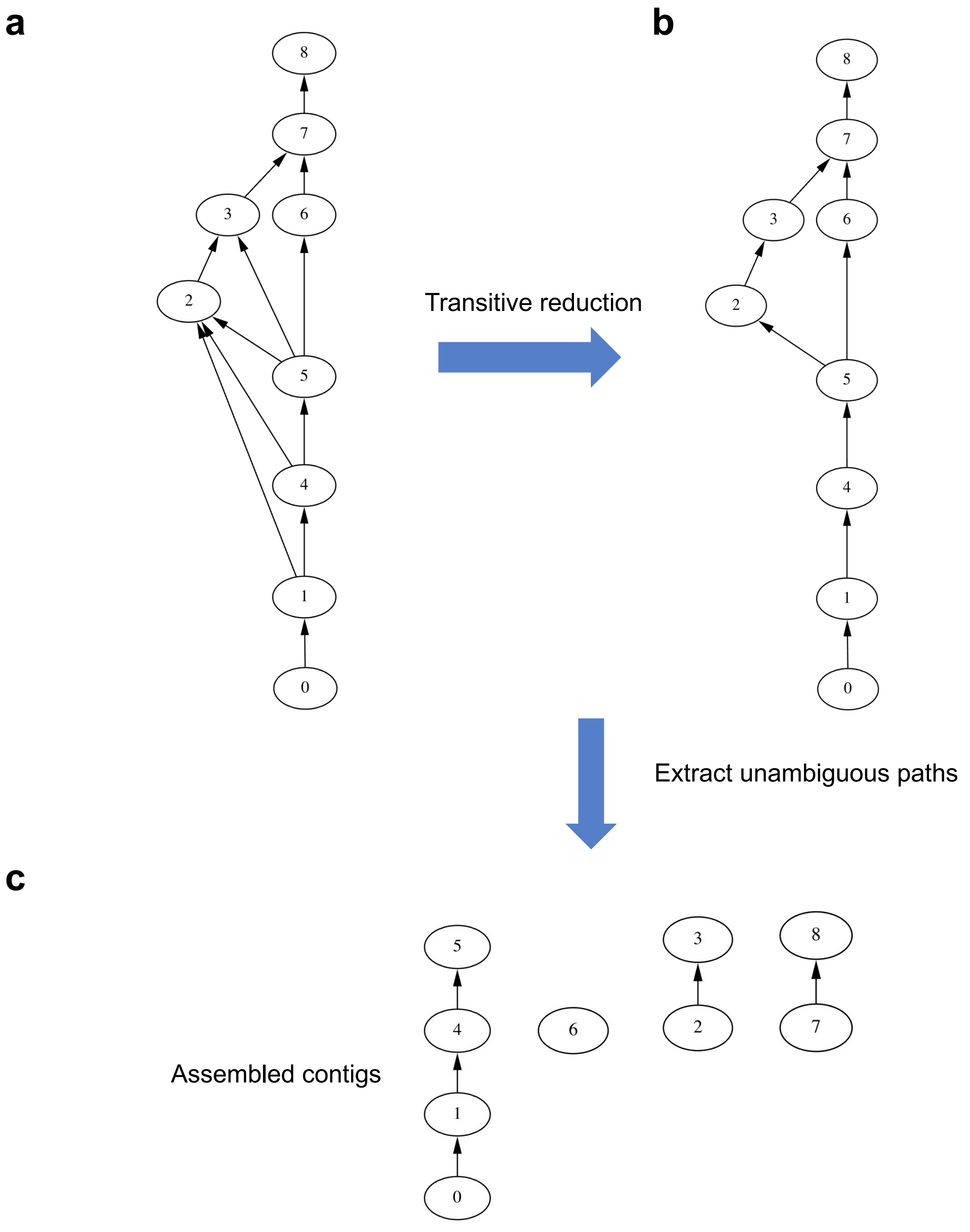

Supplementary Figure 16: **Assembling draft contigs.** **a** A demo graph obtained by overlapping draft contigs. Each vertex represents a draft contig and a edge means the two connected vertices overlap. **b** Removing redundant edges by transitive reduction. **c** Assembled contigs are obtained by extracting unambiguous paths from the graph in subfigure **b**.
