# Supplementary material for: Detecting and phasing minor single-nucleotide variants from long-read sequencing data
Source: Nat Commun. 2021 May 24;12:3032. doi: 10.1038/s41467-021-23289-4 (PMC8144375; doi:10.1038/s41467-021-23289-4)
Supplement: Supplementary file 3 — Description of Additional Supplementary Files [file 41467_2021_23289_MOESM3_ESM.pdf]

Description of Additional Supplementary Files

File Name: Supplementary Data file 1.
Description: SRA IDs of the PacBio Bordetella and E. coli data.

File Name: Supplementary Data file 2.
Description: SRA IDs of the ONT K. pneumoniae data.

File Name: Supplementary Data file 3.
Description: SRA IDs of the metagenomic data.

File Name: Supplementary Data file 4.
Description: SRA IDs of the data used to train the context effect model.

File Name: Supplementary Data file 5.
Description: The R2 of cross validation using different parameters to train the context effect model.
